# Supplementary material for: Cryptosporidium spp. and Giardia duodenalis emissions from humans and animals in the Three Gorges Reservoir in Chongqing, China
Source: PeerJ. 2020 Nov 3;8:e9985. doi: 10.7717/peerj.9985 (PMC7646300; doi:10.7717/peerj.9985)
Supplement: File S1 [file peerj-08-9985-s001.docx]

***Cryptosporidium* spp. and *Giardia*** ***duodenalis* emissions from humans and animals in the Three Gorges Reservoir in Chongqing, China**

Qian Huang^1^, Ling Yang^1^, Bo Li^1^, Huihui Du^1,2^, Feng Zhao^1^, Lin Han^1,2^, Qilong Wang^1^, Yunjia Deng^1^, Guosheng Xiao^1,2,3^, and Dayong Wang^4^

^1^ College of Biology and Food Engineering, Chongqing Three Gorges University, Wanzhou, Chongqing, China

^2^ Engineering Technology Research Center of Characteristic Biological Resources in Northeast Chongqing, Chongqing Three Gorges University, Wanzhou, Chongqing, China

^3^ Key Laboratory of Water Environment Evolution and Pollution Control in Three Gorges Reservoir, Chongqing Three Gorges University, Wanzhou, Chongqing, China

^4^ Medical School, Southeast University, Nanjing, Jiangsu, China

Corresponding Author:

Guosheng Xiao

College of Biology and Food and Engineering, Chongqing Three Gorges University, Wanzhou District, Chongqing, China

Email address: xgs03@sanxiau.edu.cn

**Supporting Information:**

**Table S1 Human and livestock densities in Chongqing in 2013.**

|  | Human population | |  | Animal population | | | | |
| --- | --- | --- | --- | --- | --- | --- | --- | --- |
| Region | Urban | Rural |  | Rabbits | Pigs | Cattle | Sheep and goats | Poultry |
| Banan District | 7.39E+05 | 2.19E+05 |  | 3.77E+05 | 5.96E+05 | 9.73E+05 | 1.57E+06 | 2.54E+06 |
| Beibei District | 5.97E+05 | 1.64E+05 |  | 4.66E+04 | 2.10E+05 | 2.57E+05 | 4.67E+05 | 7.24E+05 |
| Bishan District | 3.27E+05 | 3.65E+05 |  | 3.66E+06 | 4.02E+06 | 7.68E+06 | 1.17E+07 | 1.94E+07 |
| Changshou District | 4.69E+05 | 3.34E+05 |  | 4.46E+05 | 7.80E+05 | 1.23E+06 | 2.01E+06 | 3.23E+06 |
| Chengkou County | 5.71E+04 | 1.34E+05 |  | 4.91E+04 | 1.83E+05 | 2.32E+05 | 4.14E+05 | 6.46E+05 |
| Dadukou District | 3.18E+05 | 1.05E+04 |  | 8.17E+05 | 8.28E+05 | 1.65E+06 | 2.47E+06 | 4.12E+06 |
| Dazu District | 3.63E+05 | 3.82E+05 |  | 0.00E+00 | 3.82E+05 | 3.82E+05 | 7.63E+05 | 1.14E+06 |
| Dianjiang County | 2.68E+05 | 4.20E+05 |  | 7.70E+05 | 1.19E+06 | 1.96E+06 | 3.15E+06 | 5.11E+06 |
| Fengdu County | 2.43E+05 | 3.77E+05 |  | 6.47E+04 | 4.42E+05 | 5.06E+05 | 9.48E+05 | 1.45E+06 |
| Fengjie County | 2.90E+05 | 4.95E+05 |  | 3.41E+05 | 8.36E+05 | 1.18E+06 | 2.01E+06 | 3.19E+06 |
| Fuling District | 6.78E+05 | 4.40E+05 |  | 5.82E+05 | 1.02E+06 | 1.60E+06 | 2.63E+06 | 4.23E+06 |
| Hechuan District | 8.05E+05 | 5.23E+05 |  | 1.84E+06 | 2.36E+06 | 4.20E+06 | 6.56E+06 | 1.08E+07 |
| Jiangbei District | 7.88E+05 | 4.19E+04 |  | 0.00E+00 | 4.19E+04 | 4.19E+04 | 8.38E+04 | 1.26E+05 |
| Jiangjin District | 7.65E+05 | 5.00E+05 |  | 2.90E+06 | 3.40E+06 | 6.31E+06 | 9.71E+06 | 1.60E+07 |
| Jiulongpo District | 1.05E+06 | 1.07E+05 |  | 3.00E+04 | 1.37E+05 | 1.67E+05 | 3.04E+05 | 4.71E+05 |
| Kaizhou District | 4.73E+05 | 6.89E+05 |  | 9.24E+06 | 9.93E+06 | 1.92E+07 | 2.91E+07 | 4.83E+07 |
| Liangping District | 2.61E+05 | 4.10E+05 |  | 1.68E+05 | 5.78E+05 | 7.46E+05 | 1.32E+06 | 2.07E+06 |
| Nanan District | 7.80E+05 | 4.99E+04 |  | 0.00E+00 | 4.99E+04 | 4.99E+04 | 9.98E+04 | 1.50E+05 |
| Nanchuan District | 2.90E+05 | 2.61E+05 |  | 2.49E+05 | 5.10E+05 | 7.59E+05 | 1.27E+06 | 2.03E+06 |
| Pengshui District | 1.57E+05 | 3.68E+05 |  | 2.39E+04 | 3.92E+05 | 4.16E+05 | 8.08E+05 | 1.22E+06 |
| Qianjiang District | 1.98E+05 | 2.55E+05 |  | 2.27E+04 | 2.77E+05 | 3.00E+05 | 5.77E+05 | 8.77E+05 |
| Qijiang District | 5.85E+05 | 5.07E+05 |  | 8.26E+05 | 1.33E+06 | 2.16E+06 | 3.49E+06 | 5.65E+06 |
| Rongchang | 3.11E+05 | 3.66E+05 |  | 1.65E+06 | 2.02E+06 | 3.67E+06 | 5.69E+06 | 9.36E+06 |
| Shapingba District | 1.04E+06 | 6.73E+04 |  | 0.00E+00 | 6.73E+04 | 6.73E+04 | 1.35E+05 | 2.02E+05 |
| Shizhu District | 1.48E+05 | 2.52E+05 |  | 6.15E+06 | 6.40E+06 | 1.25E+07 | 1.89E+07 | 3.15E+07 |
| Tongliang District | 2.95E+05 | 3.42E+05 |  | 2.81E+06 | 3.15E+06 | 5.96E+06 | 9.10E+06 | 1.51E+07 |
| Tongnan District | 2.82E+05 | 3.67E+05 |  | 3.16E+05 | 6.83E+05 | 9.99E+05 | 1.68E+06 | 2.68E+06 |
| Wanzhou District | 9.53E+05 | 6.42E+05 |  | 4.26E+05 | 1.07E+06 | 1.49E+06 | 2.56E+06 | 4.06E+06 |
| Wulong District | 1.31E+05 | 2.19E+05 |  | 8.17E+05 | 1.04E+06 | 1.85E+06 | 2.89E+06 | 4.74E+06 |
| Wushan County | 1.62E+05 | 3.07E+05 |  | 8.65E+05 | 1.17E+06 | 2.04E+06 | 3.21E+06 | 5.25E+06 |
| Wuxi County | 1.19E+05 | 2.79E+05 |  | 4.22E+05 | 7.01E+05 | 1.12E+06 | 1.82E+06 | 2.95E+06 |
| Xiushan District | 1.71E+05 | 3.21E+05 |  | 1.49E+04 | 3.36E+05 | 3.51E+05 | 6.87E+05 | 1.04E+06 |
| Yongchuan District | 6.59E+05 | 4.09E+05 |  | 6.57E+06 | 6.98E+06 | 1.35E+07 | 2.05E+07 | 3.41E+07 |
| Youyang District | 1.60E+05 | 4.04E+05 |  | 3.49E+04 | 4.39E+05 | 4.74E+05 | 9.13E+05 | 1.39E+06 |
| Yubei District | 1.14E+06 | 3.21E+05 |  | 2.80E+06 | 3.13E+06 | 5.93E+06 | 9.06E+06 | 1.50E+07 |
| Yunyang County | 3.32E+05 | 5.70E+05 |  | 7.75E+05 | 1.34E+06 | 2.12E+06 | 3.46E+06 | 5.58E+06 |
| Yuzhong Distric | 6.50E+05 | 0.00E+00 |  | 0.00E+00 | 0.00E+00 | 0.00E+00 | 0.00E+00 | 0.00E+00 |
| Zhongxian County | 2.75E+05 | 4.57E+05 |  | 8.20E+06 | 8.66E+06 | 1.69E+07 | 2.55E+07 | 4.24E+07 |
| Total | 1.71E+07 | 1.19E+07 |  | 5.43E+07 | 6.67E+07 | 1.21E+08 | 1.88E+08 | 3.09E+08 |

^1^ The data was derived from Chongqing Statistic Bureau, China (http://tjj.cq.gov.cn/).

**Table S2 Data sources of variables in the GloWPa-Crypto TGR model.**

| **Variable Name (unit)** | **Symbol** | **Mean value** | **Reference** |
| --- | --- | --- | --- |
| Fraction of urban connected (%) | *F_cu_* | 77.99 | (*Sun et al., 2014*) Statistical yearbooks 2014 of Chongqing, China (http://tjj.cq.gov.cn/zwgk_233/tjnj/) |
| Fraction of urban direct (%) | *F_du_* | 22.01 | (*Sun et al., 2014*) Statistical yearbooks 2014 of Chongqing, China (http://tjj.cq.gov.cn/zwgk_233/tjnj/) |
| Fraction of rural connected (%) | *F_cr_* | 9 | 2013 China Environmental Status Bulletin (http://www.mee.gov.cn/hjzl/zghjzkgb/lnzghjzkgb/) |
| Fraction of rural diffuse (%) | *F_difr_* | 91 | 2013 China Environmental Status Bulletin (http://www.mee.gov.cn/hjzl/zghjzkgb/lnzghjzkgb/) |
| Fraction removed by wastewater treatment (%) | *F_rem_* | 50 | (*Hofstra et al., 2013*) |
| Average oocyst excretion rate (oocysts/year) | $\text{O}_{\text{p}\text{,C}}$ | $\text{1×10}^{\text{8}}$ | (*Hofstra et al., 2013*) |
| Average cyst excretion rate (cysts/year) | $O_{p,G}$ | $\text{1.58×10}^{\text{8}}$ | (*Ferguson et al., 2007; Hofstra et al., 2013*) |
| Fraction of rural resident feces applied as a fertilizer (%) | $\text{F}_{\text{s,h}}$ | 85.2 | (*Liu et al., 2019*) |
| Fraction of livestock manure applied as a fertilizer (%) | $\text{F}_{\text{s,a}}$ | 80 | (*Liu et al., 2019*) |
| Average air temperature (℃) | *T* | 19.9 | Statistical yearbooks 2014 of Chongqing, China (http://tjj.cq.gov.cn/zwgk_233/tjnj/) |
| Manure storage time (days) | $\text{t}_{\text{s}}$ | 30 | (*Liu et al., 2019*) |
| Fraction of maximum surface runoff (%) | *F_run, max_* | 50 | China Soil Database (<http://vdb3.soil.csdb.cn/>) (*Velthof et al., 2009)* |
| Reduction factor for land use | *f_lu_* | 1.0 | China Soil Database (<http://vdb3.soil.csdb.cn/>) (*Velthof et al., 2009*) |
| Reduction factor for yearly precipitation | *f_p,m_* | 0.5 | China Meteorological Data Service Center (CMDC) (<http://sthjj.cq.gov.cn/>) (*Velthof et al., 2009)* |
| Reduction factor for depth to rock | *f_rc_* | 0.8 | China Soil Database (<http://vdb3.soil.csdb.cn/>) (*Velthof et al., 2009*) |
| Reduction factor for soil type | *f_s_* | 1.0 | China Soil Database (<http://vdb3.soil.csdb.cn/>) (*Velthof et al., 2009*) |

**Table S3 The breeding days for different livestock species in Chongqing.**

| **Livestock species** | **Breeding days** |
| --- | --- |
| Rabbits | 76 |
| Boars | 183 |
| Sows and gilts for breeding | 365 |
| Beef cows | 365 |
| Dairy cows | 365 |
| Poultry | 61 |
| Sheep and goats | 243 |

^1^ These data were based on the Chongqing Ecology and Environment Bureau report (http://sthjj.cq.gov.cn/).

**Table S4 Model input for manure production and oocyst and cyst excretion rates for different livestock species.**

| **Livestock species** | **Production**  **(kg/day)** | ***Cryptosporidium***  **(^10^log oocysts·kg^-1^·d^-1^)** | ***Giardia***  **(^10^log cysts·kg^-1^·d^-1^)** | **Reference** |
| --- | --- | --- | --- | --- |
| Rabbits | 0.15 | 3.78 | 0 | (*Peng & wang, 2004; Ferguson et al., 2007*) |
| Boars | 3.5 | 5.51 | 5.72 | (*Peng & wang, 2004; Ferguson et al., 2007*) |
| Sows and gilts for breeding | 5.0 | 5.51 | 5.72 | (*Peng & wang, 2004; Ferguson et al., 2007*) |
| Beef cows | 17.5 | 3.71 | 5.23 | (*Peng & wang, 2004; Ferguson et al., 2007*) |
| Dairy cows | 25 | 3.71 | 5.23 | (*Peng & wang, 2004; Ferguson et al., 2007*) |
| Poultry | 0.12 | 6.32 | 2.88 | (*Peng & wang, 2004; Ferguson et al., 2007*) |
| Sheep and goats | 2.6 | 3.30 | 0 | (*Peng & wang, 2004; Ferguson et al., 2007*) |

**Table S5** ***Cryptosporidium* prevalence in the manure of livestock in China.**

| **Livestock species** | ***Cryptosporidium* average prevalence (%)** | ***Giardia* average prevalence (%)** |  | **References** |
| --- | --- | --- | --- | --- |
| Rabbits | 2.84 | 4.91 |  | (*Zhang et al., 2012; Qi et al., 2015; Zhang et al., 2018; Liu et al., 2019*) |
| Pigs | 18.53 | 7.16 |  | (*Wang et al., 2017; Wang et al., 2018; Liu et al., 2019*) |
| Cattle | 10.31 | 5.58 |  | (*Liu et al., 2015; Jian et al., 2018; Cui et al., 2018; Liu et al., 2019*) |
| Poultry | 8.38 | 8.38 |  | (*Liu et al., 2019*) |
| Sheep and goats | 6.30 | 5.46 |  | (*Yin et al., 2018; Chen et al., 2019; Liu et al., 2019*) |

^1^ The average prevalence of Cryptosporidiosis and Giardiasis is adapted from a systematic literature review.

^2^ The literature reviews on which the *Giardia* prevalence in the manure of livestock in China are based did not yield any results for giardiasis prevalence for poultry, we take the same values as for cryptosporidiosis prevalence.

**Table S6 Sensitivity analysis for all parameters except prevalence and excretion rates.**

| **Variable name** | **Variable changed** | **Variable baseline** | **Explanation** | **Total oocyst emissions** | **Total divided by**  **baseline run** | |
| --- | --- | --- | --- | --- | --- | --- |
| human population (*P*) | 0.8 | 1 | 20% decrease | 1.33E+15 | | 0.84 |
| human population (*P*) | 1.2 | 1 | 20% increase | 1.83E+15 | | 1.16 |
| animal numbers (*N*) | 0.8 | 1 | 20% decrease | 1.51E+15 | | 0.96 |
| animal numbers (*N*) | 1.2 | 1 | 20% increase | 1.65E+15 | | 1.04 |
| animal manure production | 0.8 | varies per animal | 20% decrease | 1.51E+15 | | 0.96 |
| animal manure production | 1.2 | varies per animal | 20% increase | 1.65E+15 | | 1.04 |
| fraction connected (urban, *F_cu_*) | 0.5 | 1 | halve | 1.92E+15 | | 1.21 |
| fraction connected (urban, *F_cu_*) | 2.0 | 1 | double | 1.39E+15 | | 0.88 |
| fraction connected (rural, *F_cr_*) | 0.5 | 1 | halve | 1.56E+15 | | 0.99 |
| fraction connected (rural, *F_cr_*) | 2.0 | 1 | double | 1.62E+15 | | 1.03 |
| Oocyst and cyst removal efﬁciencies sewage treatment (*F_rem_*) | 0.5 | 1 | halve | 1.94E+15 | | 1.23 |
| Oocyst and cyst removal efﬁciencies sewage treatment (*F_rem_*) | 2.0 | 1 | double | 8.47E+14 | | 0.54 |
| fraction of rural resident feces applied as a fertilizer (*F_s,h_*) | 0.35 | 0.85 | storage -50% | 1.51E+15 | | 0.95 |
| fraction of rural resident feces applied as a fertilizer (*F_s,h_*) | 1 | 0.85 | storage +50% | 1.60E+15 | | 1.01 |
| fraction of livestock manure applied as a fertilizer (*F_s,a_*) | 0.30 | 0.8 | storage -50% | 1.36E+15 | | 0.86 |
| fraction of livestock manure applied as a fertilizer (*F_s,a_*) | 1 | 0.8 | storage +50% | 1.66E+15 | | 1.05 |
| average air temperature (*T*) | 8.3 average  January  temperature | average annual  temperature (°C) | seasonal storage | 1.64E+15 | | 1.04 |
| average air temperature (*T*) | 31.5 average  July temperature | average annual  temperature (°C) | seasonal storage | 1.46E+15 | | 0.92 |
| manure storage time ($\text{t}_{\text{s}}$) | 15 | 30 days (1 month) | 2 weeks | 1.40E+15 | | 0.89 |
| manure storage time ($\text{t}_{\text{s}}$) | 60 | 30 days (1 month) | 2 months | 1.75E+15 | | 1.11 |
| fraction of runoff | 0.1 | 0.2 | halve | 1.35E+15 | | 0.85 |
| fraction of runoff | 0.4 | 0.2 | double | 2.04E+15 | | 1.30 |

^1^ We study the sensitivity of the GloWPa-Crypto TGR modeled *Cryptosporidium* human and livestock emissions in Chongqing to Changes in several model parameters.

^2^ Column 1 gives the variable name, column 2 is the changed value, column 3 is the standard model value and column 4 is an explanation in words. Columns 5 and 6 show the results of the model run with that particular change. Column 5 shows total oocyst emissions to surface water, and column 6 shows total oocyst emissions divided by total oocyst emissions of the baseline model run, so the relative effect of the change.

^3^ For this sensitivity analysis, the changed values were adapted from systematic literature reviews (*Vermeulen et al 2015; Vermeulen et al 2017*).

**Table S7 Sensitivity analysis for excretion rates.**

| **Variable name** | **Variable changed** | **Variable baseline** | **Explanation** | **Total oocyst emissions** | **Total divided by baseline run** |
| --- | --- | --- | --- | --- | --- |
| exc human | 7 | 8 | low (−1log) | 4.68E+14 | 0.30 |
| exc human | 9 | 8 | high (+1log) | 1.27E+16 | 8.03 |
| exc rabbits | 2.78 | 3.78 | low (−1log) | 1.58E+15 | 1.00 |
| exc rabbits | 4.78 | 3.78 | high (+1log) | 1.58E+15 | 1.00 |
| exc pigs | 4.51 | 5.51 | low (−1log) | 1.39E+15 | 0.88 |
| exc pigs | 6.51 | 5.51 | high (+1log) | 3.51E+15 | 2.22 |
| exc cattle | 2.71 | 3.71 | low (−1log) | 1.58E+15 | 1.00 |
| exc cattle | 4.71 | 3.71 | high (+1log) | 1.58E+15 | 1.00 |
| exc poultry | 5.32 | 6.32 | low (−1log) | 1.46E+15 | 0.93 |
| exc poultry | 7.32 | 6.32 | high (+1log) | 2.75E+15 | 1.74 |
| exc sheep and goats | 2.3 | 3.3 | low (−1log) | 1.58E+15 | 1.00 |
| exc sheep and goats | 4.3 | 3.3 | high (+1log) | 1.58E+15 | 1.00 |
| exc all low | varies per human and animal | varies per human and animal | low (−1log) | 1.58E+14 | 0.10 |
| exc all high | varies per human and animal | varies per human and animal | high (+1log) | 1.58E+16 | 10.00 |

^1^ Column 1 gives the variable name, column 2 is the changed value, column 3 is the standard model value and column 4 is an explanation in words. The values in columns 2 and 3 are in ^10^log oocysts·kg^-1^·d^-1^ for livestock, and ^10^log oocysts·person^-1^. yr^-1^ for humans. Columns 5 and 6 show the results of the model run with that particular change. Column 5 shows total oocyst emissions to surface water, and column 6 shows the total oocyst emissions divided by total oocyst emissions of the baseline model run, so the relative effect of the change.

^2^ First the excretion rate was changed for humans or per animal species, and the last two rows show the effect when the excretion rates are changed for humans and all animal species at the same time.

**Table S8** **Sensitivity analysis for prevalence.**

| **Variable name** | **Variable changed** | **Variable baseline** | **Total oocyst emissions** | **Total divided by baseline run** |
| --- | --- | --- | --- | --- |
| prev human | 15.00 | 10.00 | 2.20E+15 | 1.39 |
| prev rabbits | 3.40 | 2.84 | 1.58E+15 | 1.00 |
| prev pigs | 38.00 | 18.53 | 1.80E+15 | 1.14 |
| prev cattle | 29.10 | 10.31 | 1.58E+15 | 1.00 |
| prev poultry | 16.30 | 8.38 | 1.70E+15 | 1.08 |
| prev sheep and goats | 11.40 | 6.30 | 1.58E+15 | 1.00 |
| Prev all high | varies per human and animal | varies per human and animal | 2.54E+15 | 1.61 |

^1^ Column 1 gives the variable name, column 2 is the changed value and column 3 is the variable baseline. Columns 4 and 5 show the results of the model run with that particular change. Column 4 shows total oocyst emissions to surface water, and column 5 shows total oocyst emissions divided by total oocyst emissions of the baseline model run, so the relative effect of the change.

^2^ For humans, we take the prevalence increased by 50% in the sensitivity analysis (column 2). For animals, we take the highest prevalence found per animal species in this same literature review from the average prevalence of cryptosporidiosis (Table S3). We did not do a sensitivity run for the lowest observed prevalence, as this was zero in most cases and therefore not so meaningful.

^3^ First the prevalence was changed for humans or per animal species, and the last row shows the effect when the prevalence is changed for humans and all animal species at the same time.

**Table S9 Population growth, urbanization, sanitation, wastewater treatment and livestock numbers for Scenarios 1, 2 and 3 in the 2050s.**

| **Scenario features** | **2013** | **Scenario 1** | **Scenario 2** | **Scenario 3** |
| --- | --- | --- | --- | --- |
| Total population | 2.97E+07 | 2.709E+07 | 3.073E+07 | 2.709E+07 |
| Urban population (%) | 58.34 | 85.57 | 63.12 | 85.57 |
| Connected to sewer (%) |  |  |  |  |
| Urban | 77.99 | 99 | 77.99 | 99 |
| Rural | 9 | 50 | 9 | 50 |
| Sewage treatment (%) based on connected to the sewage system | | | | |
| Urban |  |  |  |  |
| Primary (removal rate: (10%) | 0 | 0 | 0 | 0 |
| Secondary (removal rate: (50%) | 77.99 | 49.50 | 77.99 | 77.99 |
| Tertiary (removal rate: (95%) | 0 | 49.50 | 0 | 0 |
| No treatment (no removal) | 22.01 | 1 | 22.01 | 22.01 |
| Rural |  |  |  |  |
| Primary (removal rate: (10%) | 0 | 0 | 0 | 0 |
| Secondary (removal rate: (50%) | 9 | 25 | 9 | 9 |
| Tertiary (removal rate: (95%) | 0 | 25 | 0 | 0 |
| No treatment (no removal) | 91 | 50 | 91 | 91 |
| Total animal number | 4.59E+08 | 6.53E+08 | 7.16E+08 | 6.53E+08 |
| Manure treatment (%) | 10 | 45 | 10 | 10 |

**Table S10** **Total *Cryptosporidium* and *Giardia* emissions per source in Chongqing for 2013 and changes for scenarios 1, 2 and 3 for 2050.**

| **Emission sources** | **2013 Total oocyst and cyst emissions**  **(oocysts and cyst/year)** | **2050 Scenario 1 changed (%)** | **2050 Scenario 2 changed (%)** | **2050 Scenario 3 changed (%)** |
| --- | --- | --- | --- | --- |
| Urban residents | 2.7E+15 | -0.43 | +0.12 | +0.25 |
| Rural residents | 4.6E+14 | -0.61 | -0.08 | +0.29 |
| Livestock | 4.9E+14 | -0.20 | +0.56 | +0.42 |

^1^ Column 1 gives the sources of emissions, column 2 is the total *Cryptosporidium* and *Giardia* emissions in 2013, column 3 is the 2050 scenario 1 change in total oocyst and cyst emissions, column 4 is the 2050 scenario 2 change in total oocyst and cyst emissions, column 5 is the 2050 scenario 3 change in total oocyst and cyst emissions.

**Table S11. Equations and parameters in calculating mean concentrations of *Cryptosporidium* oocysts and *Giardia* cysts in the TGR.**

| **No. Eq** | **Model description** | **Calculation formula** | **Parameter description** | **Parameter values** | **References** |
| --- | --- | --- | --- | --- | --- |
| 1 | Temperature-dependent survival | $\text{K}_{\text{T}}\text{=}\text{K}_{\text{4}}\text{e}^{\text{λ(}\text{T}_{\text{w}}\text{-4)}}$ | $\text{K}_{\text{4}}:$ decay rate constants at 4ºC ($\text{day}^{\text{-1}}$);  $\text{K}_{\text{T}}:$ decay rate constants at water temperature $\text{T}_{\text{w}}$ ($\text{day}^{\text{-1}}$);  $\lambda$: dimensionless modifier of temperature;  *T_w_*_:_ the TGR’s annual average temperature (℃). | $\text{K}_{\text{4}}\text{:}$ 0.0051;  $\text{λ}$: 0.18;  $\text{T}_{\text{w}}:$ 18.96. | $\text{K}_{\text{4}}:$ (*Peng et al., 2008*);  $\text{λ}$: (*Peng et al., 2008*);  $\text{T}_{\text{w}}:$ in this study. |
| 2 | Solar radiation-dependent survival | $\text{K}_{\text{R}}\text{=}\frac{\text{k}_{\text{l}}\text{ }\text{I}_{\text{A}}}{\text{k}_{\text{d}}\text{ C}_{\text{DOC }}\text{Z}}\text{ ×(1-}\text{e}^{\text{-}\text{k}_{\text{d}}\text{ C}_{\text{DOC }}\text{Z}}\text{)}$ | $\text{K}_{\text{R}}$: decay rate constant of solar radiation ($\text{day}^{\text{-1}}$);  $\text{k}_{\text{l}}$and $\text{k}_{\text{d}}$ represent proportionality constants;  $\text{I}_{\text{A}}:$ the TGR’s annual average surface solar radiation $\text{(KJ}^{\text{-2}}{\cdot\text{day}}^{\text{-1}}\text{)}$;  $\text{C}_{\text{DOC }}:$ the TGR’s annual average dissolved organic carbon concentration (${\text{mg}\text{·}\text{L}}^{\text{-1}}$) ; $\text{Z}$: the TGR’s annual average water level (m). | $\text{k}_{\text{l}}:$ 0.0004798;  $\text{k}_{\text{d}}$: 9.831;  $\text{I}_{\text{A}}:$ 564148;  $\text{C}_{\text{DOC }}:$ 3.14;  $\text{Z}$: 163. | $\text{k}_{\text{l}}$: (*Vermeulen et al., 2019*);  $\text{k}_{\text{d}}$: (*Vermeulen et al., 2019*);  $\text{I}_{\text{A}}:$ China Meteorological Data Service Center (CMDC) (<http://sthjj.cq.gov.cn/>);  $\text{C}_{\text{DOC }}:$ (*Fan et al., 2017*);  $\text{Z}$: in this study. |
| 3 | Sedimentation | $\text{K}_{\text{S}}\text{=}\frac{\text{v}}{\text{Z}}$ | $\text{K}_{\text{S}}:$ loss rate constant of sedimentation ($\text{day}^{\text{-1}}$);  *v*: settling velocity (m·$\text{day}^{\text{-1}}$);  $\text{Z}$: the TGR’s annual average water level (m). | *v*: 0.1;  $\text{Z}$: 163. | *v*: (*Vermeulen et al., 2019*);  $\text{Z}$: in this study. |
| 4 | Water residence time | $\text{t=}\frac{\text{L}}{\text{U}}\text{=}\frac{\text{L×Z×1.22Q}^{\text{0.557}}}{\text{Q}}$ | $\text{t}\text{:}$ residence time of oocyst in Chongqing section of the TGR (days);  $\text{U}$: the TGR’s flow velocity (${\text{m}\text{·}\text{s}}^{\text{-1}}$);  $\text{Q}\text{:}$ the TGR’s annual average inflow ($\text{m}^{\text{3}}\cdot\text{s}^{\text{-1}}$);  $\text{Z}$: the TGR’s annual average water level (m);  $L$: the length of the TGR in Chongqing (m). | $\text{Q}\text{:}$ 14423;  $\text{Z}$:$\text{ }$163;  $L:$ 665. | $\text{Q}\text{:}$ Changjiang [Hydrology](javascript:;) (http://www.cjh.com.cn/);  $\text{Z}$: in this study;  $L:$ (*Cheng et al., 2019*). |
| 5 | Oocyst or cyst concentrations | $\text{C=}\frac{E_{t}\text{×}\text{e}^{\text{-(}\text{K}_{\text{T}}\text{+}\text{K}_{\text{R}}\text{+}\text{K}_{\text{S}}\text{)×t}}}{Q_{s}}$  $\text{E}_{\text{t}}\text{=}\sum_{\text{k=1}}^{\text{38}} \text{E}_{\text{k}}$ | $\text{C}\text{:}$ mean concentration (oocysts and cysts·10L^-1^);  $E_{t:}$ the sum of total oocyst or cyst emissions from humans and animals in all districts or counties in Chongqing (oocysts and cysts·year^-1^);  $\text{E}_{\text{k}}\text{:}$ total oocyst or cyst emissions from humans and animals in district or county *k* (oocysts and cysts·year^-1^)  $\text{K}_{\text{T}}$, $\text{K}_{\text{R}}$ and $\text{K}_{\text{S}}$ represent loss rate constants of temperature, solar radiation, and sedimentation, respectively ($\text{day}^{\text{-1}}$);  $\text{t}$: residence time of oocyst and cysts in Chongqing section of the TGR (days);  $Q_{s}\text{:}$ the sum of the TGR’s annual inflow ($\text{Q}$) and storage capacity ($Q_{c}$) (m^3^·year^-1^). | $\text{Q}\text{:}$ 4.49×10^11^  $Q_{c}$: 2.94×10^11^ | $\text{E}_{\text{k}}\text{:}$*:* in this study  $\text{K}_{\text{T}}:$ Eq 1 in this table;  $\text{K}_{\text{R}}:$ Eq 2 in this table;  $\text{K}_{\text{S}}:$ Eq 3 in this table;  $\text{t}$: Eq 4 in this table;  $\text{Q}\text{ and }\text{Q}_{\text{c}}\text{:}$ Changjiang [Hydrology](javascript:;) (http://www.cjh.com.cn/). |

^1^ We used a hydrological model (*Vermeulen et al., 2019*) to calculated mean concentrations of *Cryptosporidium* and *Giardia* in the waters of the TGR.

References

**Chen D, Zou Y, Li Z, Wang SS, Xie SC, Shi LQ, Zou FC, Yang JF, Zhao GH, Zhu XQ. 2019.** Occurrence and multilocus genotyping of *Giardia* *duodenalis* in black-boned sheep and goats in southwestern China. *Parasites & Vectors* **12**:102 DOI: [10.1186/s13071-019-3367-1](https://doi.org/10.1186/s13071-019-3367-1).

**Cheng LD, Deng HP, He S, Gu L, Qu HH. 2019.** Distribution patterns and diversity of plant communities in fluctuating areas of Chongqing section of the Yangtze River. *Chinese Journal of Ecology* **38**(12): 3626-3634 DOI: 10.13292/j.1000-4890.201912.033. (in Chinese)

**Cui Z, Wang L, Cao L, Sun M, Liang N, Wang H, Chang Y, Lin X, Yu L, Wang R, Zhang S, Ning C, Zhang L. 2018.** Genetic characteristics and geographic segregation of *Giardia* *duodenalis* in dairy cattle from Guangdong Province, southern China. [*Infection, Genetics and Evolution*](https://www.sciencedirect.com/science/journal/15671348) **66**: 95–100 DOI: [10.1016/j.meegid.2018.09.019](https://doi.org/10.1016/j.meegid.2018.09.019).

**Fan ZW，Hao QJ，Huang Z，Chai XS，Jiang CS. 2017.** Change and influencing factors of dissolved carbon and dissolved nitrogen in water of the Three Gorges Reservoir. [*Environmental*](javascript:;) [*Sciences*](javascript:;) **38**(1): 129-137 DOI: 10．13227/j．hjkx．201606031. (in Chinese)

**Ferguson CM, Croke BF, Beatson PJ, Ashbolt NJ, Deere DA. 2007.** Development of a process-based model to predict pathogen budgets for the Sydney drinking water catchment. *Journal of Water and Health* **5**: 187-208 DOI: 10.2166/wh.2007.013b.

**Hofstra N, Bouwman AF, Beusen AH, Medema GJ. 2013***.* Exploring global *Cryptosporidium* emissions to surface water. *Science of the Total Environment* **442**: 10-19 DOI: 10.1016/j.scitotenv.2012.10.013.

**Jian Y, Zhang X, Li X, Karanis G, Ma L, Karanis P. 2018.** Prevalence and molecular characterization of *Giardia* *duodenalis* in cattle and sheep from the Qinghai-Tibetan rcn Plateau Area (QTPA), northwestern China. [*Veterinary Parasitology*](https://www.sciencedirect.com/science/journal/03044017) **250**: 40–44 DOI: [10.1016/j.vetpar.2017.12.001](https://doi.org/10.1016/j.vetpar.2017.12.001).

**Liu G, Su Y, Zhou M, Zhao J, Zhang T, Ahmad W, Lu H, Jiang N, Chen Q, Xiang M, Yin J. 2015.** Prevalence and molecular characterization of *Giardia* *duodenalis* isolates from dairy cattle in northeast China. [*Experimental Parasitology*](https://www.sciencedirect.com/science/journal/00144894) **154**: 20–24 DOI: [10.1016/j.exppara.2015.03.020](https://doi.org/10.1016/j.exppara.2015.03.020).

**Liu W, An W, Jeppesen E, Ma J, Yang M, Trolle D. 2019***.* Modelling the fate and transport of *Cryptosporidium*, a zoonotic and waterborne pathogen, in the Daning River watershed of the Three Gorges Reservoir Region, China. *Journal of Environmental Management* **232**: 462-474 DOI: 10.1016/j.jenvman.2018.10.064.

**Peng L, Wang DY. 2004.** Estimation of annual quantity of total excretion from livestock and poultry in Chongqing Municipality. *Transactions of the Chinese Society of Agricultural Engineering* **20**: 288-292 (in Chinese).

**Peng X, Murphy T, Holden NM. 2008.** Evaluation of the effect of temperature on the die-off rate for *Cryptosporidium parvum* oocysts in water, soils, and faeces. *Applied and environmental microbiology* **74**(23):7101-7107 DOI: 10.1128/AEM.01442-08.

**Qi M, Xi J, Li J, Wang H, Ning C, Zhang L. 2015.** Prevalence of Zoonotic *Giardia* *duodenalis* Assemblage B and First Identification of Assemblage e in Rabbit Fecal Samples Isolates from Central China. [*Journal of Eukaryotic Microbiology*](http://xueshu.baidu.com/usercenter/data/journal?cmd=jump&tn=SE_baiduxueshu_c1gjeupa&ie=utf-8&sc_f_para=sc_hilight=publish&sort=sc_cited&wd=journaluri%3A%287ea82024c99e26c%29%20Journal%20of%20Eukaryotic%20Microbiology) **62**: 810–814 DOI: [10.1111/jeu.12239](https://doi.org/10.1111/jeu.12239).

**Sun Y, Zhang F, Hu HY, Niu ZB. 2014**. Statistical analysis of influent quality characteristics of municipal wastewater treatment plants in Chongqing. *Environmental Science and Technology* **37**: 397-402 (in Chinese).

**Velthof GL, Oudendag D, Witzke HP, Asman WA, Klimont Z, Oenema O. 2009***.* Integrated assessment of nitrogen losses from agriculture in EU-27 using MITERRA-EUROPE. *Journal of Environmental Quality* **38**: 402-417 DOI: 10.2134/jeq2008.0108.

**Vermeulen LC, Benders J, Medema G, Hofstra N. 2017.** Global *Cryptosporidium* loads from livestock manure. environmental science and technology **51**: 8663-8671 DOI: 10.1021/acs.est.7b00452.

**Vermeulen LC, Kraker JD, Hofstra N, Kroeze C, Medema G. 2015**. Modelling the impact of sanitation, population growth and urbanization on human emissions of *Cryptosporidium* to surface waters—a case study for Bangladesh and India. *Environmental Research Letters* **10**: 094017 DOI: 10.1088/1748-9326/10/9/094017.

**Vermeulen LC, van Hengel M. Kroeze C, Medema G, Spanier JE, van Vliet MTH. 2019.** *Cryptosporidium* concentrations in rivers worldwide. *Water Research* **149**: 202-214 DOI: 10.1016/j.watres.2018.10.069.

**Wang H, Zhang Y, Wu Y, Li J, Qi M, Li T, Wang J, Wang R, Zhang S, Jian F, Ning C, Zhang L. 2018.** Occurrence, Molecular characterization, and assessment of zoonotic risk of *Cryptosporidium* spp., *Giardia* *duodenalis*, and *enterocytozoon* *bieneusi* in pigs in Henan, Central China. [*Journal of Eukaryotic Microbiology*](https://www.researchgate.net/journal/1066-5234_Journal_of_Eukaryotic_Microbiology)  **65**: 893–901 DOI: [10.1111/jeu.12634](https://doi.org/10.1111/jeu.12634).

**Wang SS, Yuan YJ, Yin YL, Hu RS, Song JK, Zhao GH. 2017.** Prevalence and multilocus genotyping of *Giardia* *duodenalis* in pigs of Shaanxi Province, northwestern China. *Parasites and Vectors* **10**: 1–8 DOI: [10.1186/s13071-017-2418-8](https://doi.org/10.1186/s13071-017-2418-8).

**Yin YL, Zhang HJ, Yuan YJ, Tang H, Chen D, Jing S, Wu HX, Wang SS, Zhao GH. 2018.** Prevalence and multi-locus genotyping of *Giardia* *duodenalis* from goats in Shaanxi province, northwestern China. [*Acta Tropica*](https://www.sciencedirect.com/science/journal/0001706X) **182**: 202–206 DOI: 10.1016/j.actatropica.2018.03.013.

**Zhang W, Shen Y, Wang R, Liu A, Ling H, Li Y, Cao J, Zhang X, Shu J, Zhang L. 2012.** *Cryptosporidium* cuniculus and *giardia* *duodenalis* in rabbits: Genetic diversity and possible zoonotic transmission. *PLoS* *One* **7**: 13–17 DOI: [10.1371/journal.pone.0031262](https://doi.org/10.1371/journal.pone.0031262).

**Zhang X, Qi M, Jing B, Yu F, Wu Y, Chang Y, Zhao A, Wei Z, Dong H, Zhang L. 2018.** Molecular characterization of *Cryptosporidium* spp., *Giardia* *duodenalis*, and *enterocytozoon* *bieneusi* in rabbits in Xinjiang, China. [*Journal of Eukaryotic Microbiology*](http://xueshu.baidu.com/usercenter/data/journal?cmd=jump&tn=SE_baiduxueshu_c1gjeupa&ie=utf-8&sc_f_para=sc_hilight=publish&sort=sc_cited&wd=journaluri%3A%287ea82024c99e26c%29%20Journal%20of%20Eukaryotic%20Microbiology) **65**: 854–859 DOI: [10.1111/jeu.12629](https://doi.org/10.1111/jeu.12629).
